# Prevalence of CBAVD in azoospermic men carrying pathogenic *CFTR* mutations ‐ Evaluated in a cohort of 639 non‐vasectomized azoospermic men

**DOI:** 10.1111/andr.12925

**Published:** 2020-11-07

**Authors:** Jens Fedder, Mette W. Jørgensen, Birte Engvad

**Affiliations:** ^1^ Centre of Andrology & Fertility Clinic Odense University Hospital Odense Denmark; ^2^ Institute of Clinical Medicine University of Southern Denmark Odense Denmark; ^3^ Department of Clinical Genetics Lillebaelt Hospital Vejle Denmark; ^4^ Department of Pathology Odense University Hospital Odense Denmark

**Keywords:** azoospermia, *CFTR* mutations, CBAVD, CUAVD, testis

## Abstract

**Background:**

Men with obstructive azoospermia (OA) due to impaired development of the genital tract often carry at least one Cystic Fibrosis Transmembrane Conductance Regulator *CFTR* mutation.

**Objective:**

To determine the frequency of Congenital Bilateral Absence of *Vas deferens* (CBAVD) in men with azoospermia carrying *CFTR* gene mutations.

**Materials and methods:**

Non‐vasectomized men with azoospermia referred to our andrological center were consecutively included. All men underwent palpation of the scrotal parts of the *Vasa deferentia*, ultrasonography of the testicles and hormone profile, and genetic analyses. Testicular biopsy was usually performed. A panel of 32 of the most important *CFTR* mutations was examined from genomic DNA isolated from blood lymphocytes. Either multiplex PCR analysis or a next‐generation sequencing technique was performed.

**Results:**

Among the 639 men with azoospermia, 69 (10.8%) had at least one *CFTR* mutation. Of the 43 patients with at least one of the two *CFTR* mutations, *ΔF508* and *R117H*, 19 (44.2%) showed CBAVD, 2 (4.7%) Congenital Unilateral Absence of Vas deferens (CUAVD), and 22 (51.2%) presence of the scrotal parts of the *Vasa deferentia*. In contrast, only 1/21 men (4.8%) with an isolated *IVS8‐5T* variant showed CBAVD. Among the further 20 men with an isolated IVS8‐5T variant, 11 had a history of cryptorchidism. Among the 570 men without *CFTR* mutations, CBAVD was found in only two men and CUAVD in one. FSH level was higher and testicular volume lower in men with present *Vasa deferentia* compared to those without (*P* < .001; Student's t test). Thirty‐one men with either *ΔF508* or *R117H* mutations, or both, had a testicular biopsy. Motile spermatozoa were found in 100% of 16 cases with CBAVD but in only 6 out of 15 cases with present *Vasa deferentia* (*P* < .01; Fisher's exact test).

**Discussion and conclusions:**

CBAVD was found in ~ 44% of men with ΔF508/R117H mutations. The data may support that CFTR mutations might affect male fertility through other mechanisms than obstruction of the genital tract. For a practical, clinical purpose analysis for only *ΔF508*, *R117H* and *IVS8‐5T* seems sufficient until further research shows anything else.

## 1 INTRODUCTION

During more than 50 years it has been known that the majority (97%) of men with Cystic Fibrosis (CF) exhibit Congenital Bilateral Absence of *Vas Deferens* (CBAVD).^1^ While only persons homozygous for two severe pathogenic Cystic Fibrosis Transmembrane conductance Regulator (*CFTR*) mutations develop the disease CF, men with *CFTR* compound heterozygoti or a single pathogenic *CFTR* exon mutation, in some cases combined with an intron 8 5T splice variant, may have CBAVD as an isolated phenotype without symptoms from the respiratory tract and gastrointestinal system.^2^ *CFTR* mutations are also suggested to modulate the epididymal microenvironment and to inhibit spermatogenesis and capacitation,^3^ but the exact mechanism is unclear.

The *CFTR* gene, located on chromosome 7 (q 31.2), consists of 27 exons and 26 introns. The *CFTR* gene encodes a channel protein that enables chloride passage through apical cell membranes. The well known *CFTR* mutations *ΔF508* ^4^, ^5^ and *R117H* are located in exon 10 and exon 4, respectively. In addition to the exon mutations, intron mutations – or variants – are important. Thus, the polythymidine sequence located in the splicing acceptor site of intron 8 is found in three variants with 9, 7, or 5 thymidines: the *IVS8‐9T*, *IVS8‐7T*, and *IVS8‐5T*, respectively. The *9T* and *7T* alleles predominantly generate normal mRNA transcripts, whereas the *5T* variant affects exon 9 resulting in reduced levels of normal mRNA. The *5T* allele is thus considered as a mild pathogenic mutation with incomplete penetrance. The relative contributions of different *CFTR* mutations and variants show considerable variation between different populations,^6^, ^7^, ^8^ the *ΔF508* being the dominant CFTR mutation in Caucasians.^2^ In Caucasians, CFTR exon mutations occur with a prevalence of around 4% in the background population.^9^

Many studies have documented an extremely high prevalence of *CFTR* mutations in men with obstructive azoospermia (OA) and CBAVD. *CFTR* mutations occur in up to 40% of non‐vasectomized men with OA.^10^ In a metaanalysis including 38 studies, 78% of men with CBAVD carried one or two (a severe and a mild or two mild) *CFTR* mutations.^2^

Conversely, the risk to develop CBAVD when carrying *CFTR* mutations is insufficiently studied. A reasonable cause may be that most andrologists and fertility clinics only perform *CFTR* gene analysis in men with OA and maybe even CBAVD/ Congenital Unilateral Absence of Vas deferens (CUAVD). Thus, the present cohort is unique since all men with azoospermia were tested for the clinically most relevant *CFTR* mutations, in addition to careful clinical examination, including palpation of the scrotal parts of the *Vasa deferentia*.

As it is unclear if *CFTR* mutations may also decrease fertility by mechanisms other than genital tract obstruction,^3^ the first aim of this study was to estimate the prevalence of CBAVD in men with azoospermia carrying one or two *CFTR* mutations. While the prevalence of *CFTR* *in* CBAVD has been analyzed in other studies, the converse consideration thus is quite new. The second aim was to clarify if analysis for *CFTR* mutations should be performed in men with non‐obstructive azoospermia (NOA) and OA without CBAVD/CUAVD. This second aim is not quite new,^11^, ^12^ but conclusions are still very controversial. Thus, as mentioned above, *CFTR* mutations are suggested to inhibit spermatogenesis.^3^ To answer this question, we compare FSH levels, testicular and ejaculate volumes, testicular histology, and rates of presence of motile, testicular spermatozoa between azoospermic *CFTR* carriers with and without CBAVD. These data will be analyzed in relation to clinical data, to evaluate if other clinical conditions can explain possible differences.

## 2 MATERIALS AND METHODS

### 2.1 Patients

Non‐vasectomized men with azoospermia, defined as at least two consecutive ejaculates without spermatozoa in the pellet after centrifugation, have been evaluated using an almost identical evaluation program since 1997. Until May 1., 2011, patients were examined in the Fertility Clinic, Braedstrup Hospital.^13^ Hereafter patients were examined in the Centre of Andrology and Fertility Clinic, OdenseUniversityHospital, as the authorities selected this center to be one of two highly specialized centers of Andrology in Denmark. The included azoospermic men represent the majority of azoospermic men evaluated for infertility in Western Denmarkin the period mentioned.

Thus, since about 1% of the male population is expected to have azoospermia,^14^ an incidence of almost 150 new cases is expected in Western Denmark every year. We have about 75 new cases referred every year. The explanations of the difference may be, that some may choose treatment with donor semen without further examination, choose to live without having children, and a few with a simple etiology may have been treated in a fertility clinic not specialized in andrology. In addition to vasectomized men, men with aspermia due to ejaculatory disorders such as anejaculation or retrograde ejaculation^15^ were not included in this study.

### 2.2 Clinical examination program

Men with azoospermia underwent a detailed history interview and careful clinical examination including scrotal palpation of the testicles, the *Epididymides*, and the *Vasa deferentia*. History interview included questions about cryptorchidism, genital tract infections, cancer, and other former or present diseases, trauma, irradiation, chemotherapy, working health, heated seats, sauna, medications, smoking, and alcohol use.^16^ Additionally, ultrasonography of the scrotum, including measurement of the testicular volumes, was performed as described by Fedder.^16^ These clinical examinations, including ultrasonography, were in all cases performed before genetic and hormonal analyses. Hormone profiles, including follicular stimulating hormone (FSH), luteinizing hormone (LH), testosterone, prolactine, and thyroid‐stimulating hormone (TSH), karyotype analyses, and analyses for Y microdeletions and the clinically most relevant *CFTR* mutations were in every case performed. Furthermore, in recent years, inhibin‐B, anti‐Müllerian hormone (AMH), and serum‐estradiol were also determined.

Men with elevated FSH levels, small testicular volumes, and testicular histology showing hypospermatogenesis, maturation arrest, or Sertoli cell‐only syndrome (SCO) were categorized as having non‐obstructive azoospermia, while men with normal FSH levels, normal‐sized testicles, and normal testicular tissue at histological examination were categorized as having obstructive azoospermia.

### 2.3 Analyses of CFTR mutations

Genomic DNA was extracted from blood lymphocytes and analyzed according to routine analyses. During the entire period, all men with azoospermia were examined for the three exon *CFTR* mutations: *ΔF508* (exon 10), *R117H* (exon 4), and *394delTT* (exon 3), and furthermore for the polythymidine *5/7/9T* variants in *IVS8* (intron 8). Since 2008, the PCR technique used was changed to an extended multiplex PCR followed by an oligonucleotide‐ligation analysis on the amplified DNA, and since 2018 the PCR has been replaced by a next‐generation sequencing (NGS) examining the same 32 mutations. After DNA fragmentation (Covaris), the NGS panel genes were amplified and the libraries sequenced (Illuminis MiSeq DNA sequenator).

Thus, since 2008 the routine analysis has included the following 32 CFTR mutations: *ΔF508*, *394delTT*, *R117H*, *S549N*, *A455E*, *R347H*, *S549R*, *R347P*, *R553X*, *W1282X*, *G551D*, *2184delA*, *R334W*, *V520F*, 2789+5G>A, *1078delT*, *I507del*, *1898+1G>A*, *3849+10kbC>T*, *621+1G>T*, *R1162X*, *3876delA*, *711+1G>T*, *N1303K*, *1717‐1G>A*, *G85E, 3659delC*, *G542X*, *2183AA>G*, *3905insT*, *R560T* and *3120+1G>A*. And in addition analysis for the *IVS8‐5/7/9T* was continously performed.

### 2.4 Karyotyping and examination for Y microdeletions

These analyses were performed as described elsewhere.^10^ After harvesting and culture of blood lymphocytes and blockade of mitosis, the metaphase chromosomes were stained with Giemsa (G). Based on the resulting G banding it was possible to identify each chromosome and construct the karyotype.

For the detection of Y microdeletions, based on extracted genomic DNA, at least two sequence‐tagged sites (STSs) for each of the three regions: AZFa, AZFb, and AZFc were used for detection of deletions in the respective regions. As controls, STSs on Yp and Yq were used.^10^

### 2.5 Testicular biopsies and testicular histology

Unless the patient had Klinefelter's syndrome or primarily asked for treatment with donor semen, diagnostic testicular biopsy was usually performed. Sperm retrieval was usually performed as TEsticular Sperm Extraction (TESE) using a 14 gauge TruCut needle^17^ For further details see Fedder et al.^10^, ^18^ A piece of testicular tissue from each biopsy was immediately squeezed in our IVF lab (as described in detail in ^19^and examined for the presence of motile and immotile sperm under microscope. Another piece of tissue was fixed in Bouins fixative, and sections stained with hematoxylin and eosin (HE), periodic acid‐Schiff reagent (PAS), and Verhoeffs elastin stain, respectively, for histological examination. Testicular histological patterns were divided into: (a) normal spermatogenesis where spermatozoa were found in the majority of seminiferous tubules, (b) maturation arrest when germ cell maturation was blocked at a specific level, (c) hypospermatogenesis when all steps of the spermatogenesis were present but at a reduced level and (d) Sertoli cell‐only syndrome when practically all the seminiferous tubules consisted only of Sertoli cells.^20^, ^21^

### 2.6 Ultrasonography of the kidneys in azoospermic men with CUAVD

Since an increased frequency of renal aplasia has been suggested in the literature in patients with CUAVD,^22^ the azoospermic men with CUAVD underwent ultrasonography to visualize the kidneys.

### 2.7 Statistics

Comparisons on parametric data such as FSH, testicular volumes, and ejaculate volumes were calculated using Student's t test after verification of normality. Comparisons on categorical variables such as numbers with testicular spermatozoa or normal histology in relation to *CFTR* mutations or presence of CBAVD and implantation and pregnancy rates were performed using the Fisher's exact test.

### 2.8 Approvals

The study was approved by the Danish Patient Safety Authority (journal nr 3‐3013‐2503/1) and the Danish Data Protection Agency (journal nr 18/18147).

## 3 RESULTS

Among this cohort of azoospermic men, more than 80% showed NOA. One‐hundred and twenty‐five men (19.6%) showed KS or Y microdeletions, and in addition 173 (27.1%) had a history of cryptorchidism. Less frequent occurring causes included hypogonadotrophic hypogonadism and chemotherapy due to malignancy.

As shown in Table 1, the proportion of men with NOA has weakly increased during the 21‐year period that patients were included. During the whole period, men with all kinds of azoospermia from our region were included without selection. However, during the final half of the period, an increasing number of selected men with azoospermia were referred to micro‐TESE from all regions of Denmark. This reflects an increasing proportion of men with, for example, Klinefelter's syndrome in our cohort. This condition may have contributed to the increase in men with NOA and resulted in slightly decreased proportion of men with *CFTR* mutations.

**Table 1:** Distribution of azoospermic men with ^a^non‐obstructive azoospermia, a history of cryptorchidism, Klinefelter's syndrome, Y microdeletions, or ^b^ *CFTR* mutations during the period 1997‐2018. Significance levels between the periods 1997‐2011 vs. 2011‐2018 were calculated with the χ^2^‐test considering *P* < .05 as significance level

	Period 1 1997‐2011	Period 2 2011‐2018	Significance level	Patients in total 1997‐2018
Total No. of Azoospermic men	289	350	639	
Non‐obstructive azoospermia	81.7% (236)	84.9% (297)	NS	83.4% (533)
History of cryptorchidism	30.8% (89)	24.0% (84)	NS	27.1% (173)
Klinefelter's syndrome	9.7% (28)	18.6% (65)	*P* = .002	14.6% (93)
Y microdeletions	4.8% (14)	5.1% (18)	NS	5.0% (32)
*CFTR* mutations	12.8% (37)	9.1% (32)	NS	10.8% (69)

^a^ Non‐obstructive azoospermia.

^b^ Cystic Fibrosis Transmembrane conductance Regulator.

The first (until 2008) 195 azoospermic men (30.5% of the cohort) were analyzed for only four *CFTR* mutations (*ΔF508*, *R117H*, *394delTT*, *IVS8‐5T*), while the extended panel of 32 mutations was only available for the final 444 men (69.5% of the cohort). Inside this cohort, 69 (10.8%) of the 639 men carried at least one *CFTR* mutation. At least one of the important *ΔF508* and *R117H* mutations were found in 43 cases (6.7% of the total cohort), either isolated, in combination with each other, or in combination with the *IVS8‐5T* variant (Table 2).

**Table 2:** Relationship between the presence of ^a^ *CFTR* mutations/variants and presence of the scrotal parts of the *Vasa deferentia* in 639 consecutive men with azoospermia included in the study

± *CFTR* mutations/ variants	Congenital Bilateral Absence of *Vas deferens* (CBAVD)	Congenital Unilateral Absence of *Vas deferens* (CUAVD)	Scrotal parts of both V*asa deferentia* present
*ΔF508* and/or *R117H* exon mutations	19	2	22
Other *CFTR* exon mutations	1	0	4
*IVS8‐5T* variant without exon mutation	1	0	20
No ^a^ *CFTR* gene abnormalities detected	2	1	567

^a^ Cystic Fibrosis Transmembrane conductance Regulator.

*CFTR* mutations other than *ΔF508*, *R117H,* and *IVS8‐5T* were found in only 5 (1.1%) of the 444 azoospermic men exposed to the big panel analyzing for 32 *CFTR* mutations. Among these 5 men, only 1 showed CBAVD, while other explanations of the infertility should probably be found in the other 4 men.

In a couple of cases the family, including the parents of the man, was examined for the presence of *CFTR* mutations, and in these cases we are sure that the two *CFTR* mutations are located on two different alleles (“*trans*” position). In other cases, for example, men with both *ΔF508* and *R117H*, we find it unprobably that both mutations should be located on the same allel (“*cis*” position). With the assumption that *CFTR* mutations in men with more than one *CFTR* mutation are in all cases located in a “*trans*” position (compound heterozygoti), the allel frequency of *CFTR* mutations (*IVS8* variants excluded) can be calculated to 4.4% (*ΔF508*: 2.5%, *R117H*: 1.4%, other *CFTR* mutations: 0.5%).

The majority of the 69 non‐vasectomized men with azoospermia carrying *CFTR* mutations were Danish; only 11 (15.9%) of the men were of other nationalities. Thus, of the 25 men carrying an isolated *ΔF508* mutation with or without an accessory *IVS8‐5T* variant (Table 3), 3 were from Great Britain, Romania, or Iceland, while 22 (88%) were of Danish ethnicity. Among the 10 men with an isolated *R117H* mutation (± IVS8‐5T), 2 were from United States or Bosnia‐Herzegovina, while 8 (80%) were of Danish origin. Of 21 men with an isolated *IVS8‐5T* variant, 4 (19%) were from the Arabic world^3^ or Asia.^1^ Finally, among only 5 men in the group with “other single‐gene mutations” (Tables 2 and 3), 2 (40%) were from Syria and had intact *Vasa deferentia* at scrotal examination (Table 3). All men with azoospermia and suggested compound heterozygosity for *CFTR* mutations in this cohort were of Danish ethnicity.

**Table 3:** FSH, testis volume (both testicles), ejaculate volume, and the chance of retrieving motile spermatozoa from testicular biopsy arranged according to respective ^a^ *CFTR* gene mutations and presence or absence of the *Vasa deferentia*. Values are given as mean ± SD (range)

CFTR mutation	*Vas deferens*	Number	FSH (IU/L)	Testicular volume (mL) (both testicles)	^c^Ejaculate volume (mL)	Percentage (number) with motile spermatozoa
*ΔF508*	^b^VD present	13	13.1 ± 8.8 (3.0‐30)	12.7 ± 7.3 (1.8‐23.9)	2.3 ± 2.0 (0.2‐7.5)	44% (4/9)
	CBAVD	8	4.6 ± 1.4 (2.5‐6.6)	24.8 ± 8.0 (13.3‐36.0)	0.6 ± 0.5 (0.3‐1.6)	100% (8/8)
*R117H*	VD present	5	13.1 ± 6.3 (5.6‐21)	12.3 ± 3.5 (8.7‐16.1)	2.6 ± 1.3 (0.5‐4.0)	25% (1/4)
	CBAVD	4	7.2 ± 2.8 (4.4‐11)	39.6 ± 17.1 (25.5‐62.4)	0.9 ± 0.5 (0.4‐1.5)	100% (4/4)
*IVS8‐5T*	VD present	20	21.8 ± 16.0 (1.0‐63)	13.9 ± 8.4 (1.3‐29.5)	3.6 ± 1.5 (1.1‐6.0)	71% (10/14)
	CBAVD	1	2.3	22.3	0.4	100% (1/1)
Other single gene mutations	VD present	4	10.4 ± 12.0 (2.3‐28)	13.7 ± 15.6 (1.3‐36.5)	1.9 ± 1.7 (0.2‐3.5)	33% (1/3)
	CBAVD	1	4.1	40.0	1.3	100% (1/1)
*ΔF508* + *R117H*	VD present	2	4.2 ± 1.3 (3.3‐5.1)	24.2 ± 12.0 (15.7‐32.6)	1.4 ± 1.1 (0.6‐2.2)	50% (1/2)
	CBAVD	5	3.1 ± 2.1 (1.7‐6.9)	38.3 ± 28.0 (8.6‐83.9)	1.0 ± 0.7 (0.05‐2.0)	100% (4/4)
*ΔF508* + *IVS8‐5T*	VD present	2	2.3 ± 0.3 (2.1‐2.5)	36.0 ± 20.5 (21.0‐50.0)	0.6 ± 0.4 (0.3‐0.8)	100% (2/2)
	CUAVD	1	7.1	16.4	0.3	100% (1/1)
	CBAVD	1	2.5	50.0	0.3	100% (1/1)
*R117H* + *IVS8‐5T*	CBAVD	1	2.7	39.5	‐	‐ 100% (1/1)
*394delTT* + *R117H*	CUAVD	1	3.8	23.7	2.0	100% (1/1)

Note

FSH was higher in present ID compared to CBAVD (all *CFTR* mut.: *P* = .0003; *ΔF508* mut.: *P* = .02; *R117H* mut.: *P* = .12, Student's t test). Testicular volume (both testicles) was lower in present VD compared to CBAVD (all *CFTR* mut.: *P* = .0000006; *ΔF508* mut.: *P* = .002; *R117H mut*.: *P* = .02, Student's t test). Ejaculate volume was higher in present VD compared to CBAVD (all *CFTR* mut.: *P* = .00002; *ΔF508* mut.: p=(<)0.05; *R117H* mut.: p=(<)0.05, Student's t test). Motile testicular spermatozoa were found significantly more frequently in men with CBAVD compared to men with present VD (*P* = .0002, Fisher's exact test).

^a^ Cystic Fibrosis Transmembrane conductance Regulator.

^b^ *Vasa deferentia*.

^c^ Six men distributed over the groups below unable to ejaculate.

Among 43 azoospermic men with at least one of the two most frequent CFTR exon mutations, *ΔF508* and *R117H*, 19 (44.2%) showed CBAVD, 2 (4.7%) Congenital Unilateral Absence of *Vas deferens* (CUAVD), and 22 (51.2%) presence of the scrotal parts of the *Vasa deferentia*. In contrast, CBAVD or CUAVD without an associated *CFTR* gene mutation were only found in 2 and 1, respectively, of the 639 men (~0.5%). Only 1 of 21 men (4.8%) with a probably isolated *IVS8‐5T* variant showed CBAVD, while the remaining ~ 95% with isolated *IVS8‐5T* showed normal presence of the scrotal parts of the *Vasa deferentia*. For the few other single gene or compound heterozygotes, the statistical power is insufficient to draw conclusions according to the risk of having CBAVD/CUAVD (Table 2).

Remarkably, FSH levels were higher (*P* = .0003, t test; Table 3) and testicular volumes (both testicles) lower (*P* = .0000006, t test; Table 3) for men with specific *CFTR* mutations if the scrotal parts of the *Vasa deferentia* were present compared to CBAVD (Table 3). In addition, the percentages of men with testicular spermatozoa were higher in the subgroups with CBAVD (Table 3). Thus, for azoospermic men with *ΔF508* and *R117H* mutation, isolated or in combination, motile testicular spermatozoa were found in 100% of 16 men with CBACD undergoing testicular biopsy compared to only 6 of 15 with present *Vasa deferentia* (*P* = .0002, Fisher's exact test; Table 3). These findings were furthermore supported by the histological patterns observed in testicular biopsies. Not all men had testicular biopsy, for example, two men with *ΔF508* and present *Vasa deferentia* also had KS. In men with CBAVD, normal testicular tissue was found in all cases, while affected spermatogenesis (hypospermatogenesis, maturation arrest, or Sertoli cell‐only syndrome) was found in 76% of 34 cases with presence of both *Vasa deferentia* (*P* = .00001, Fisher's exact test; Table 4). However, since normal testicular tissue was found in 8 men with both *Vasa deferentia* present, an undetected obstruction might be suggested in these cases.

**Table 4:** Testicular histological patterns shown in relation to ^a^ *CFTR* gene mutations/variants and presence or absence of the *Vasa deferentia*. Affected spermatogenesis (hypospermatogenesis, maturation arrest, or Sertoli cell‐only syndrome (SCO)) was significantly associated with the presence of the *Vasa deferentia* (*P* = .00001, Fisher's exact test)

*CFTR* mutation arrest	CBAVD				Both *Vasa deferentia* present			
	Normal testicular tissue	Hypospermatogenesis	Maturation arrest	SCO	Normal testicular tissue	Hypospermatogenesis	Maturation arrest	SCO
*ΔF508*	8	‐	‐	‐	2	4	‐	3
*R117H*	4	‐	‐	‐	1	1	‐	2
*IVS8‐5T*	1	‐	‐	‐	1	9^b^	1	4
Other single gene mutations	1	‐	‐	‐	1	‐	‐	2
*ΔF508* + *R117H*	4	‐	‐	‐	2	‐	‐	‐
*ΔF508* + *IVS8‐5T*	1	‐	‐	‐	1	‐	‐	‐
*R117H* + *IVS8‐5T*	1	‐	‐	‐	‐	‐	‐	
No mutation/variant	‐	‐	‐		‐			

^a^ Cystic Fibrosis Transmembrane conductance Regulator.

^b^ One patient had testicular biopsy before referral to our department, and immediate examination for living spermatozoa (Table 3) was not performed in this case.

While *IVS8‐5T* was not associated to CBAVD, surprisingly 11 of 20 cases (55%) had an orchiopexy during childhood (Table 5; *P* = .004, chi‐square test) suggesting a possible association between IVS8‐5T and cryptorchidism. The coincidence between *IVS8‐5T* and chemotherapy or KS (Table 5) in a few cases probably is random.

**Table 5:** Clinical characteristics of the 20 non‐vasectomized, azoospermic men presenting both *Vasa* deferentia in addition to the *IVS8‐5T* variant. Histological findings shown for the 15 men having a diagnostic testicular biopsy performed

Clinical characteristics			Histological pattern		
Main clinical characteristic	Additional clinical characteristics	Normal testicular tissue	Hypospermatogenesis	Maturation arrest	SCO
^a^Orchiopexia in childhood: 11 cases^a^	Beckwith‐Wiedemann syndrome: 1 case Delayed puberty: 1 case	1	7	‐	3
Intense chemotherapy due to leukemia or cancer in the chest: 2 cases	‐	‐	1	‐	‐
Klinefelter's syndrome: 1 case	‐	‐	‐	‐	‐
Pathological level of small hypereckogenic foci in the testicles at ultrasonography: 2 cases	‐	‐	1	‐	‐
No pathological clinical findings: 4 cases	Inversion chromosome 1	‐	1^b^	‐	1

^a^ Orchiopexia was bilateral in 8 cases

^b^ The one with the chromosome 1 inversion

Coincidental diagnoses were relatively infrequent in *CFTR* exon mutation carriers. Two *ΔF508* carriers with present *Vasa deferentia* had a KS karyotype (pure 47,XXY) and two *CFTR* carriers a Y microdeletion (AZFbc in a *ΔF508* and AZFc in a *R117H* carrier). In addition, 3 *ΔF508* carriers with present *Vasa deferentia* were treated for cryptorchidism in childhood (2 with orchiopexia), but also 2 CBAVD patients (1 with a *R117H* and 1 with a *c3659del* mutation) and 1 CUAVD patient (*R117H + 394delTT* carrier) were treated for cryptorchidism in childhood (2 with orchiopexia). In addition, one CBAVD patient carrying a *ΔF508* mutation had taken anabolic steroids for 5‐6 years. Beyond the mentioned cases, genetic abnormalities or history of cryptorchidism or genital tract infections were in no case detected. The frequency of possible additional explanations for the infertility did not differ significantly between *CFTR* patients with or without CBAVD/CUAVD.

Men with CBAVD showed lower ejaculate volumes compared to azoospermic men with present *Vasa deferentia* (*P* = .00002, Fisher's exact test; Table 3). Renal aplasia could not be detected with ultrasonography in any of the three men with CUAVD.

## 4 DISCUSSION

Traditionally azoospermia has been divided into obstructive (OA) and non‐obstructive azoospermia (NOA). This division not always is quite unambiguous, since a few patients may be a little difficult to categorize, and some men may develop impaired spermatogenesis secondary to OA.^23^ However, due to our usual criteria the proportion of azoospermic men with NOA exceeds 80% and may have slightly increased during the study period (Table 1). This may be due to referral of an increased number of men with KS (*P* = .002, χ^2^‐test), while a trend for reduced proportions of azoospermic men with a history of cryptorchidism or *CFTR* mutations was observed (Table 1). One explanation might be that more men with NOA, including KS, are referred due to improved treatments, including m‐TESE. It cannot be excluded that a little higher number of men with OA or good prognosis NOA are now treated in peripheral fertility clinics around us. Anyway, the proportion of men with NOA has been relatively constant during the 20‐year long study period. We therefore suggest our cohort to be representative for men with azoospermia in general.

### 4.1 Main findings/What is new?

In this study including 639 consecutive men with azoospermia, one or two *CFTR* mutations/variants were detected in 69 (10.8%). Of those only 21 (30.4%) had CBAVD and 2 (2.9%) CUAVD. CBAVD was found in ~ 44% of *ΔF508/R117H* carriers but only in ~ 5% of patients with an isolated *IVS8‐5T* variant. CBAVD or CUAVD without an associated *CFTR* mutation was found in only 3 cases (0.5%) in this study. *CFTR* mutation carriers without CBAVD showed higher FSH levels, lower testicular volumes, and a lower frequency of testicular spermatozoa.

While the prevalence of *CFTR* mutations in men with OA or CBAVD has been analyzed in several studies, we demonstrate for the first time which prevalence of CBAVD can be expected in azoospermic men with *CFTR* mutations. The study also confirms that sperm retrieval is a successful treatment in men with CBAVD, since motile testicular spermatozoa were found in 100% of 16 men with CBAVD undergoing testicular biopsy. Since the prevalence of *CFTR* mutations other than *ΔF508*, *R117H*, and *IVS8‐5T* seems extremely low, the study raises doubt about the benefit of extending the *CFTR* analysis from these three analyses to analysis of 32 mutations – at least from a simple clinical point of view.

### 4.2 Strengths

One strength of this study is that it was performed on a consecutively collected material of men with azoospermia without selection, although an increasing rate of men with Klinefelter's syndrome was apparent due to the highly specialized functions, including m‐TESE, performed in our center. A significant strength in this study is that clinical examination of the presence of the scrotal parts of the *Vasa deferentia* as well as examination of the *CFTR* mutation status was performed in all patients with azoospermia and that all patients were examined by an experienced andrologist (JF).

### 4.3 Limitations

The inclusion of an increasing number of men with Klinefelter's syndrome may have resulted in slightly decreased proportion of men with *CFTR* mutations over time, which might be considered a minor weakness of the study. Furthermore, it would have been a strength of the study, if all the men with azoospermia routinely had been examined with transrectal ultrasonography (TRUS) to detect if the seminal vesicles were normal, enlarged, rudimentary, or absent. Thus, deficiencies in the genital tract, although the scrotal parts of the *Vasa deferentia* are present, may have been missed in some cases. Furthermore, informations about the size(s) of the seminal vesicles could have been added, if TRUS had been routinely performed during the study. Finally, it may seem to be a weakness, that the *CFTR* mutation analysis performed by the clinical genetic department has been extended during the whole study period. However, the most important *CFTR* mutations/variants: *ΔF508*, *R117H*, and *IVS8‐5T* were examined in all cases.

### 4.4 Prevalence

In this study, the prevalence of *CFTR* mutations and variants in men with azoospermia without CBAVD/CUAVD (7.5%) and of *CFTR* exon mutations alone (4.2%, Table 1) only slightly exceeds the expected prevalence, since the prevalence of *CFTR* exon mutations in the general population is 3%‐4%.^9^

The *ΔF508* mutation is the most prevalent mutation, particularly in Caucasians,^2^ where it is the most prevalent CFTR mutation found in CBAVD.^24^ Within 50 Iranian men with CBAVD, *ΔF508* was found in 16% ^6^ and among 60 Indian CAVD patients (do not distinguish between CBAVD and CUAVD) *ΔF508* was found in 26.7%.^7^ In contrast, *ΔF508* mutations were found in 9.4% of 53 Iranian men with non‐CBAVD obstructive azoospermia^25^ and in 11.6% of 60 Indian men with non‐CBAVD obstructive azoospermia.^26^ *ΔF508* mutations are rare in China.^8^, ^27^

The *R117H* mutation is not very frequent globally ^24^ but, in accordance with this study, it may be more frequent in the Western world.^28^ The *IVS8‐5T* variant seems to be prevalent not only in Caucasians but also worldwide.^2^

### 4.5 Possible mechanisms

One explanation for the higher prevalence of *CFTR* mutations/variants in non‐CBAVD men with azoospermia might be that the genital tract is obstructed in locations other than the scrotal parts of the *Vasa deferentia*. Thus, autopsy of men with the disease Cystic Fibrosis (CF) has shown that the retroperitoneal segments of the *Vasa deferentia* tended to be the most deficient.^1^, ^29^ Furthermore, the seminal vesicles are usually absent or rudimentary and the ejaculatory ducts absent or without lumen.^1^, ^29^ Similar findings could be expected in azoospermic men with *CFTR* mutations, although they do not have CF.

The mechanisms by which *CFTR* mutations lead to CBAVD are not completely known. However, CBAVD due to *CFTR* mutations is associated with high prevalence of atrophy or absence of the epididymides and seminal vesicles,^30^ which may explain a reduced ejaculate volume, since the major contribution to the seminal fluid comes from the seminal vesicles.

It has been found that the *CFTR* gene, which encodes the chloride channel, can be detected in the epididymides and *Vasa deferentia*.^31^ The *CFTR* protein is located in the apical membranes of the secretory epithelial cells.^31^ Dysregulation of the *CFTR* gene may affect the epididymal microenvironment through abnormal electrolyte concentrations and secretion of high viscous fluids, which may obstruct the male genital tract downstream: Tizzano et al^31^ suggested that the anatomical aberrations in the male genital tract in men carrying *CFTR* mutations occur as a consequence of production of this viscous material in the epididymides rather than as a primary congenital, anatomical abnormality of the *Vasa deferentia* and the remaining genital tract.

It is a great question whether the azoospermia can be explained by other factors than the *CFTR* mutation in *CFTR* carriers with present *Vasa deferentia*. And if the *CFTR* mutations could affect the spermatogenesis, it is a mystery why the spermatogenesis seems normal in the men with CBAVD, while our histological findings indicate severe inhibition of the spermatogenesis in *CFTR* mutation carriers with present *Vasa deferentia*. As mentioned in the result section, we have been able to find possible coincidental explantions in only a few cases with *CFTR* mutations and present *Vasa deferentia*. Therefore, it cannot be excluded that the slightly increased prevalence of *CFTR* mutations in azoospermic men without CBAVD is due to inhibition of fertility by other mechanisms than genital tract obstruction in some of these *CFTR* mutation carriers. Chen et al^3^ provided evidence that *CFTR* mutations may affect the epididymal microenvironment and inhibit spermatogenesis and sperm capacitation in men with present *Vasa deferentia*.

Anyway, it is surprising that the majority of *IVS8‐5T* carriers showed hypospermatogenesis, maturation arrest, or Sertoli cell‐only syndrome, although testicular spermatozoa were detected. Thus, CBAVD was only found in 1 of 21 azoospermic men carrying the *IVS8‐5T* variant. The findings may be explained by the high frequency of history of cryptorchidism among the *IVS8‐5T* patients. This finding may be due to a heterogenous structure where small foci with sperm production are present, although severe inhibition of spermatogenesis is evident. This is in accordance with what have been found in azoospermic men with a history of cryptorchidism in former studies.^13^ Therefore, it may be suggested that *IVS8‐5T* may have a negative effect, directly or indirectly, on spermatogenesis despite being considered as a mild *CFTR* mutation.

### 4.6 Possible effects on fertility

From minor studies, it may be suggested that testicular spermatozoa retrieved from men with NOA using microdissection TESE have a reduced potential in fertilizing oocytes compared to ejaculated donor spermatozoa.^32^ The current data on fertility are very scarce, and this study has not sufficient power to conclude, that the fertilizing capacity of the spermatozoa from *CFTR* mutation carriers with present *Vasa deferentia* is reduced compared to *CFTR* mutation carriers with CBAVD. However, in an Australian study a 17% implantation rate was obtained after transfer of embryos fertilized with fresh, epididymal, or testicular spermatozoa retrieved from men with CBAVD.^33^ Unfortunately, Phillipson et al^33^ did not have sufficient power to distinguish between use of epididymal and testicular spermatozoa. Unless reconstructive surgery of the genital tract might be considered, it is always worth to consider retrieval of spermatozoa from the epididymis to avoid the risk of testicular traumas.^34^ However, in patients with *CFTR* mutations and CBAVD the testicles use to be of normal size, and therefore, it is usually very easy to perform a testicular needle biopsy with a very low risk of complications.

In addition, in a clinical study in which epididymal spermatozoa from 63 patients with surgically confirmed congenital absence of *Vas deferens* (64% with a CFTR mutation) were used for in vitro fertilization of oocytes, *CFTR* mutations were associated with low fertilization and pregnancy rates.^35^ Immediately, it may be suggested that the reason may be that the epididymal spermatozoa are “old,” since they have not been able to leave the epididymis due to obliteration of the genital tract.^36^ However, since the 21 men with an isolated *ΔF508* mutation showed particularly low fertilization and pregnancy rates compared to other *CFTR* mutations, the effect of *ΔF508* was suggested to be moderated by other CFTR mutations present in patients who are compound heterozygotes. Another explanation may be that patients with apparently isolated *ΔF508* mutations might have another undetected *CFTR* mutation.^35^ It is not possible to repeat this study to observe the ability of the spermatozoa to penetrate the oocyte investment and fertilize the oocyte, since the majority of patients currently undergo IntraCytoplasmic Sperm Injection (ICSI) using spermatozoa retrieved from one of the testicles.

### 4.7 Association with other genes

As mentioned in the introduction the *IVS8‐5T* variant shows variation in penetrance, which may explain the reasons for variation observed in spermatogenesis in this study. It appears that *IVS8‐5T* may affect sperm production than rather resulting in CBAVD – at least in our population. However, the incomplete penetrance of the polythymidine allele *5T* seems to be associated with other polymorphic loci. Thus, the number of TG repeats in the (TG)m locus located in intron 8 (like the polythymidine sequence), affects the amount of *CFTR* gene transcripts produced.^37^ Furthermore, the *M470V* (1540A/G) polymorphism in exon 10 affects CFTR function. *CFTR* genes containing the V allele (*V470*) have lower functional ability compared to *CFTR* genes with the M allele (*M470*). Therefore, men with a *5T‐TG12‐V470* haplotype have a much higher risk of having CBAVD.^27^, ^37^ In this study, *IVS8‐5T* appeared to affect spermatogenesis rather than resulting in CBAVD. Future research is required to examine if variations in the number of TG repeats in intron 8 or *M470V* polymorphism in exon 10 may explain this discrepancy.

In this study, only three men with azoospermia (~0.5%) were diagnosed with CBAVD or CUAVD without detection of a *CFTR* mutation. In other studies, it has not been possible to detect *CFTR* mutations or variants in more than 20% of CBAVD patients.^2^ Since more than 2000 *CFTR* mutations and variants are known, CBAVD or CUAVD in some cases may be due to an undetected *CFTR* mutation. However, recently X‐linked mutations causing CBAVD were detected.^38^ These mutations are found in the Adhesion G protein‐coupled Receptor G2 gene, *ADGRG2*, which is localized on the X chromosome (Region p 22.13).^39^ There may be considerable variation between different populations, but *ADGRG2* variants may cause CBAVD in a proportion of men without *CFTR* mutations. *ADGRG2* variants have not been found associated with renal abnormalities.^38^

### 4.8 Relationship to urinary tract malformations

An association between CBAVD/CUAVD and urinary tract malformations has been described in literature.^40^ Although renal abnormalities such as unilateral renal agenesis have been described in men with CBAVD but without detectable *CFTR* mutations,^40^, ^41^ renal agenesis is particularly frequent in men with CUAVD.^22^, ^42^ Thus, Casals et al^20^ found renal anomalies in 41% 10 of 24 men with CUAVD compared to 5.4% of men with CBAVD. That is the reason that we only performed renal ultrasonography in the three patients with CUAVD, and renal abnormalities could not be detected in any of these three men. Since renal abnormalities occur less frequently in CBAVD/CUAVD patients with *CFTR* mutations compared to CBAVD/CUAVD patients without *CFTR* mutations,^22^, ^24^, ^40^ it may be suggested that renal abnormalities coexisting with CBAVD/CUAVD in men without CFTR mutations may be due to a non‐*CFTR* mechanism affecting major parts of the Wolffian ducts. In recent studies, it has been suggested that the polythymidine sequence and the numbers of TG repeats in exon 8 and the *M470V* polymorphism in exon 10, in addition to male reproductive ability, may also influence the risk of having renal abnormalities.^43^ Furthermore, also *EDNRA* and *TGF‐β1* gene polymorphisms seem to affect the incidence of CBAVD as well as development of the kidneys, seminal vesicles, epididymides, and ejaculatory ducts in men with azoospermia carrying *CFTR* mutations.^7^

## 5 CONCLUSIONS AND FUTURE DIRECTIONS

In conclusion, CBAVD was found in ~ 44% of a relatively unselected population of azoospermic men with *ΔF508*/*R117H* mutations. In addition, since normal testicular tissue was found in 8 men with present *Vasa deferentia*, it may be suggested that an undetected obstruction may be present in some cases. However, the present data also supports the hypothesis that *CFTR* mutations might affect male fertility through mechanisms other than obstruction of the male genital tract. Interestingly, we found a very high association between presence of the *CFTR* intron variant *IVS8‐5T* and a history of cryptorchidism requiring orchiopexia, which has to be further investigated in up‐coming studies. Furthermore, it will be interesting to study whether the number of TG repeats in exon 8 and the *M470V* polymorphism in exon 10 affects testicular histology and spermatogenesis in men with the *IVS8‐5T* variant.

From a practical clinical point of view, the benefit by extending the *CFTR* analysis from *ΔF508*, *R117H*, and *IVS8‐5T* to 32 mutations was very modest. Thus, it may be considered to apply the big panel with 32 mutations to only men with OA or maybe even only men with CBAVD/CUAVD, until further research shows if also other categories of infertile men should be examined for CFTR mutations and for which? Future research will determine whether analysis of sudden *CFTR* mutations (eg, the *IVS8‐*5T mutation) should also be extended to other categories of infertile men, for example, men with severe oligozoospermia and a history of cryptorchism or patients with reduced ejaculate volume or very viscous semen.

## CONFLICT OF INTERESTS

The authors declare no conflict of interest.

## AUTHOR'S CONTRIBUTIONS

JF was responsible for the concept, and he performed history interview and careful clinical examination including ultrasonography of the scrotum and testicular biopsy on all patients. He ordered blood samples and wrote the manuscript draft. MWJ performed *CFTR* mutation analyses, and BE performed histological examination of testicular biopsies. All authors approved the final version of the manuscript.
